# Immune function biomarkers in children exposed to lead and organochlorine compounds: a cross-sectional study

**DOI:** 10.1186/1476-069X-4-5

**Published:** 2005-04-14

**Authors:** Wilfried Karmaus, Kevin R Brooks, Thomas Nebe, Jutta Witten, Nadia Obi-Osius, Hermann Kruse

**Affiliations:** 1 Department of Epidemiology, Michigan State University, B601 West Fee Hall, East Lansing, MI 48824, USA; 2 Central Laboratory, University Hospital Mannheim, Germany; 3 Ministry of Social Welfare Hesse, Department of Health, Wiesbaden, Germany; 4 Epidemiological Working Group of the Ministry of Environment and Health and the Institute for Mathematics and Data Management in Medicine, University Hospital Hamburg-Eppendorf, Germany; 5 Institute of Toxicology, Christian-Albrecht University, Kiel, Germany

## Abstract

**Background:**

Different organochlorines and lead (Pb) have been shown to have immunomodulating properties. Children are at greater risk for exposure to these environmental toxicants, but very little data exist on simultaneous exposures to these substances.

**Methods:**

We investigated whether the organochlorine compounds (OC) dichlorodiphenylethylene (DDE), hexachlorobenzene (HCB), hexachlorocyclohexane (γ-HCH), the sum of polychlorinated biphenyls (ΣPCBs) and Pb were associated with immune markers such as immunoglobulin (Ig) levels, white blood cell (WBC), counts of lymphocytes; eosinophils and their eosinophilic granula as well as IgE count on basophils. The investigation was part of a cross-sectional environmental study in Hesse, Germany. In 1995, exposure to OC and Pb were determined, questionnaire data collected and immune markers quantified in 331 children. For the analyses, exposure (OC and Pb) concentrations were grouped in quartiles (γ-HCH into tertiles). Using linear regression, controlling for age, gender, passive smoking, serum lipids, and infections in the previous 12 months, we assessed the association between exposures and immune markers. Adjusted geometric means are provided for the different exposure levels.

**Results:**

Geometric means were: DDE 0.32 μg/L, ΣPCBs 0.50 μg/L, HCB 0.22 μg/L, γ-HCH 0.02 μg/L and Pb 26.8 μg/L. The ΣPCBs was significantly associated with increased IgM levels, whereas HCB was inversely related to IgM. There was a higher number of NK cells (CD56+) with increased γ-HCH concentrations. At higher lead concentrations we saw increased IgE levels. DDE showed the most associations with significant increases in WBC count, in IgE count on basophils, IgE, IgG, and IgA levels. DDE was also found to significantly decrease eosinophilic granula content.

**Conclusion:**

Low-level exposures to OC and lead (Pb) in children may have immunomodulating effects. The increased IgE levels, IgE count on basophils, and the reduction of eosinophilic granula at higher DDE concentrations showed a most consistent pattern, which could be of clinical importance in the etiology of allergic diseases.

## Background

Environmental toxicants such as organochlorine compounds (OC) and lead (Pb) may alter immune responses. There is a paucity of studies reporting associations between organochlorine [1-4] and lead [5-8] exposures and immune function biomarkers in children.

We conducted a large-scale environmental study of second-grade school children in three regions south of the Federal State of Hesse, Germany in 1995. Two of the regions are situated in the Rhine Valley with low mountains on both sides. One of these areas with several municipalities is located within a 10 km radius around an industrial waste incinerator and other industries, such as chemical plants. One plant was associated with dichlorodiphenylethylene (DDE), hexachlorobenzene (HCB), and hexachlorocyclohexane (γ-HCH) pollution [9]. The other region, also industrial, is 15 km north (downwind) of the incinerator. Both Rhine valley regions are also intensively used for the production of vegetables. The third study region is located in low mountains (about 0.4 km above sea level) that separate it from the industrial area. Blood concentrations of PCBs were shown to be higher in children living close to the toxic waste incinerator [10]. Results on PCBs and thyroid hormones, chromium and lymphocytes, DDE and breastfeeding and asthma have been published elsewhere [4,11-15].

Considering infection and atopic disorder in children, we have previously shown an association between DDE blood levels; asthma and one immunoglobulin (Ig), namely IgE [4]. However, the potential effects of organochlorines on other Igs and cellular defense were not reported. Hence, the focus of this paper is to investigate the impact of organochlorine compounds and Pb on humoral immune markers and cell-mediated immune responses. Specifically, for immune responses we focus on leukocytes, lymphocytes, B-cell, T-cells and their subsets. Assuming a concurrent effect of OC on immune markers, we conducted cross-sectional analyses of the data from the first of three surveys conducted in 1994/1995, 1996, and 1997. Only the first investigation included an extensive clinical assessment of immune markers.

## Methods

### Study population

After obtaining approval from the Data Protection Agency of Hamburg, Germany, the Ministry of Cultural Affairs of Hesse, Germany, and the local school committees, we invited the parents of 1,091 second-grade school children in 18 townships to participate in our study. We obtained informed consent from all participating parents, according to the requirements of the Ethical Committee of the Board of Physicians, the Helsinki Declaration, and the Data Protection Agency of the State of Hamburg. We asked each parent to allow their child to participate in phlebotomy only when passive smoking in the private household did not exceeded 10 cigarettes per day during the previous 12 months.

### Questionnaires

We used four self-administered parental questionnaires in the survey: one regarding the living condition and nutrition of the family, one each for the mother and the father, and one regarding information on the child. Duration of breastfeeding was recorded in weeks of total and in weeks of exclusive nursing. Environmental tobacco smoke (ETS) was graded as smoking in the child's home in the previous 12 months (no cigarettes, 1–10 cigarettes, 11–20 cigarettes, 21–30 cigarettes, more than 30 cigarettes per day). We recorded age, gender, and the number of infections, defined as cold, coughing, and sore throat with or without fever in the last 12 months (none, less than 5, 5–10, more than 10).

### Laboratory analyses of blood samples

One parent accompanied each child in the medical examination. For blood sampling, we used the 'Vacutainer System' (Becton, Dickinson & Company, San José, California,). Approximately 25 mL were drawn and separated into different aliquots. Immunoglobulin (Ig) E in serum was quantified at the Medical, Alimentary and Veterinary Institute for Research Middle Hesse, Division of Human Medicine, Dillenburg, Germany, using a florescence-immunoassay (CAP, Pharmacia, Uppsala, Sweden). To determine levels of specific IgE against inhalant allergens (aeroallergens), we incubated serum with immunocaps containing a mixture of aeroallergens and determined the reactivity using a fluorescence measurement (UNICAP Pharmacia, Uppsala, Sweden). Results from this method were provided in semi-quantitative format. We also measured IgA, G, and M by laser immunonephelometry (Dade Behring, Liederbach, Germany). The results for IgA, G and M were provided in mg/dL and for IgE in kU/L serum. Triglycerides and cholesterol were measured on a clinical chemistry analyzer according to IFCC methods (Hitachi 717, Boehringer Mannheim).

### Leukocyte subsets

We collected 8 mL of blood in tubes containing EDTA and mixed them to prevent clotting. This aliquot was transported to the Central Laboratory of the University Clinic of Mannheim and analyzed on the same day. We used 200 μL of blood for the automated differential (laser-based hematology analyzer CD3500, Abbott Diagnostics, Santa Clara, California), and 100 μL for each of the nine three-color test tubes analyzed by flow cytometry (FACScan, Becton, Dickinson, & Company, San José, California, equipped with a 488 nm air-cooled argon ion laser). Eosinophils were determined according to their specific depolarisation characteristics and their eosinophilic granula content by the intensity of light scatter by flow cytometry. Basophils were identified by their high IgE density on the cell surface using immunofluorescence with a Phycoerythrin (PE) labeled anti-IgE antibody.

We used monoclonal antibodies directed against specific cell surface antigens to differentiate cell populations by multicolour immunofluorescence. Three antibodies were simultaneously applied with the fluorochrome combination FITC/PE/PE-Cy5. CD4/CD8/CD3 was used to detect absolute number of lymphocytes, T-helper cells and cytotoxic T-cells; CD19/CD5/IGE was used to differentiate B-cell subsets and basophils; CD3/CD16 and CD56/CD57 were used for natural killer cells. CD45RO defines memory T-helper cells. The CD nomenclature assigns the antibodies to clusters of differentiation, according to the International Workshop on Human Leukocyte Differentiation Antigens [16].

### Organochlorine compounds (OC) in blood

OC including eight PCB congeners (101, 118, 138, 153, 170, 180, 183, 187), DDE, HCB, and three HCH congeners (α-, β- and γ) were determined (in μg/L) at the Institute of Toxicology, University of Kiel, Germany. OC were analyzed in 5 mL samples of whole blood by high resolution gas chromatography (HRGC, Model 3400 by Varian Inc., Palo Alto, California) with a 63Ni-electron-capture-detector. The detection limit (DL) (two times the signal/low-noise ratio) was 0.02 μg/L for β- and γ-HCH, DDE and each PCB congener, and 0.01 μg/L for HCB and α-HCH. For extraction and clean-up procedures, we used florisil and n-hexane for elution (9 g florisil were deactivated with 3% H_2_O and placed in a chromatography column 22 mm in diameter and 48 mm in length). The capillary column amounted to 30 mm in length and 0.25 mm in diameter; nitrogen was used as a carrier gas. We determined the PCB congeners by retention times on the chromatograms and identified them by comparison with known standards. Additionally, we tested reliability with gas chromatography-mass spectroscopy (GC/MS). The laboratory successfully participated in nationwide quality assessments for the determination of these OC.

### Lead in blood

Lead (Pb) analysis was done at the Institute of Toxicology, University of Kiel, Germany. The determination in whole blood samples was by flow injection atomic absorption spectroscopy (Perkin Elmer) after adding 0.1% Triton-X-1-solution and 15 mol nitric acid to from a solution. This solution was then centrifuged at 3000 rpm. The DL for Pb was 9 μg/L (48 nmol/l; atomic weight: 207.19).

### Data analyses

Since the data for leukocytes (WBC) and their subsets (lymphocytes and eosinophils), immunoglobulins, DDE, PCB congeners, HCB, γ-HCH and Pb were not normally distributed, the geometric mean, 5-, 95-percentiles are provided. In order to obtain a multivariate normal distribution, we log-transformed the number of cells and immunoglobulins before testing associations with possible predictors by multiple linear regression models.

All statistical analyses were performed using SAS software [17]. We calculated the sum of the PCB congeners (ΣPCBs = sum of seven congeners, the congener PCB101 was not detected). For descriptive purposes, we substituted values of OC below detection limit with one half of the detection limit. The statistical procedure (PROC RANK) was used to group exposure variables into quartiles (DDE, PCBs, HCB and Pb) or tertiles (γ-HCH). All observations below the detection limit were part of the lowest level group (reference). To account for the influence of lipids on the concentration of OC, we controlled for the sum of triglycerides and cholesterol in the regression analyses. Further steps were taken to determine whether our results were different when lipids were represented as sum of triglycerides and cholesterol as opposed to triglycerides and cholesterol as individual variables.

We used linear regression models (PROC GLM) with immune markers as dependent variables and all organochlorine compounds and lead as independent variables in each model. We also controlled for potential confounders (age, gender, environmental tobacco smoke (ETS), number of infections during the last 12 months, and lipid concentration). Information on passive smoking (ETS) in the child's home in the previous 12 months was divided into four categories (no cigarettes, 1–10 cigarettes, 11–20 cigarettes, 21 cigarettes per day and more). For the number of infections we considered four categories (none, less than 5, 5–10, more than 10). Age of the child was divided into three groups; 7, 8 and 9–10 years.

From the results of the regression analyses, we calculated adjusted geometric means for leukocyte subsets and immunoglobulins for increasing categories of exposure. T-tests were used to compare the statistical effect of higher exposure group to the lowest (reference).

**Figure 1:**
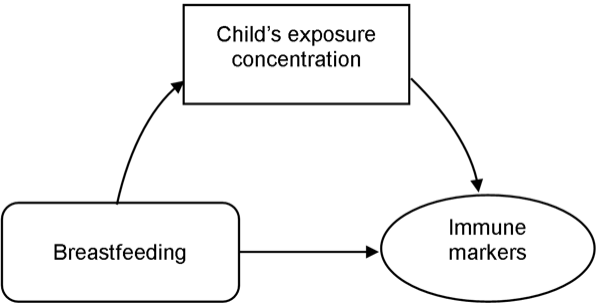
Diagramatic representation of the breastfeeding, childhood exposures and immune markers associations

Since one major route of exposure to the pollutants analyzed is breast feeding [18-21] and breastfeeding provides passive immunity [22-24], immune markers and pollutants could be spuriously correlated if breast feeding is not controlled for. However, this triangle (Figure 1) cannot be tested with linear regression models, as intervening variables do not qualify as confounders [25]. Controlling will reduce the initial association between the risk factor and the marker, as one causal chain is split into two associations. Thus, we explored the relationship between childhood breastfeeding (total duration of breastfeeding in weeks), the concentration of OC, and immune response by path analysis [26], using the CALIS procedure SAS Institute [17].

## Results

The proportion of participation was 61.5 % (671 of 1091). We obtained blood samples from 350 children, conducted OC and Pb analyses on 343 samples, and quantified immunoglobulins in 340. Overall, information (i.e., questionnaires, exposure biomarkers, and immune markers) was available for 331 children. Fewer girls than boys participated in phlebotomy; and 96 % of the children were 7 to 8 years of age (Table 1). Due to the inclusion criterion for blood sampling (passive smoking of less than 10 cigarettes in the child's home), the prevalence of passive smoking was also lower in the group with phlebotomy than in the total group (Table 1). Nevertheless, the fact that parents were separated or divorced and shared cohabitation for their child, resulted in a re-assessment of the passive smoking status after phlebotomy. Eligibility was determined on the information provided by one parent (mother or father) for their household. In the case of separate dwellings, we re-assessed the exposure by taking the average number of cigarettes smoked in both homes. As a consequence, 26 (7.9%) children who were exposed to more than 10 cigarettes per day at home had a phlebotomy and were included in the analyses.

**Table 1:** Descriptive characteristics of the study cohort.

		Total group	Subgroup with OC and immune markers
		(N = 671)	(n = 331)

		%	%

**Boys**		53.1	56.8
**Age**	7 years	45.8	46.2
	8 years	50.2	50.2
	9–10 years	4.1	3.6
**Passive smoking in the child's home during the last 12 months **(cigarettes per day)			
	None	52.2	66.5
	1–10	23.4	24.8
	11–20	14.3	5.3
	more than 30	10.1	2.4
**Number of infections during the last 12 months**			
	None	6.0	5.7
	1 to < 5	74.7	74.8
	5 to 10	17.2	17.4
	more than 10	2.1	2.1
**Duration of total breastfeeding **(weeks)			
	0	19.1	15.1
	1 to < 5	7.9	15.4
	5 – 8	12.5	12.1
	9–12	10.6	11.8
	more than 12	34.7	41.1
	Missing	5.2	4.5
**Serum cholesterol concentration**			
(mean, 5–95%-value, mg/dL)			186 (143–235)
**Triglyceride concentration**			
(mean, 5–95%-value, mg/dL)			130 (53–262)

For γ-HCH, 27.7 % of the observations were below the detection limit, 2.9% for Pb, whereas none for DDE and HCB. At least one of seven PCB congeners was detected in each sample. Whole blood concentration for the sum of PCB congeners (118, 138, 153, 170, 180, 183, 187), HCB and of Pb showed a decline with increasing age (Table 2). DDE, PCB, and HCB concentrations were lower in children with higher passive smoking exposure. Regarding infections, lead concentration was higher in children with more than 10 infections during the last 12 months, whereas DDE concentration was lower in this group (Table 2).

**Table 2:** Geometric mean and 5-, 95% values for whole blood OC and Pb by covariates.

	Category (n)	DDE (μg/L)	Sum of PCBs (μg/L)	HCB (μg/L)	γ-HCH (μg/L)	Pb (μg/L)
Gender	Girls (143)	0.32 (0.13 – 1.07)	0.43 (0.16 – 1.39)	0.21 (0.11 – 0.48)	0.02 (0.01 – 0.06)	25.4 (11.0 – 4 3.8)
	Boy (188)	0.31 (0.13 – 0.96)	0.54 (0.19 – 1.66)	0.23 (0.11 – 0.54)	0.02 (0.01 – 0.04)	27.8 (14.8 – 48.2)
Age-groups	7 years (153)	0.32 (0.13 – 0.97)	0.54 (0.18 – 1.90)	0.23 (0.11 – 0.56)	0.02 (0.01 – 0.06)	27.3 (13.9 – 48.2)
	8 years (166)	0.31 (0.13 – 0.98)	0.47 (0.18 – 1.29)	0.21 (0.11 – 0.48)	0.02 (0.01 – 0.05)	26.4 (10.7 – 47.8)
	9–10 years (12)	0.31 (0.20 – 0.84)	0.33 (0.10 – 0.99)	0.17 (0.10 – 0.46)	0.02 (0.01 – 0.06)	25.4 (16.6 – 39.4)
Passive smoking in the child's home during the last 12 months (cigarettes per day)	None (220)	0.35 (0.14 – 1.08)	0.57 (0.21 – 1.70)	0.24 (0.11 – 0.55)	0.02 (0.01 – 0.06)	26.5 (10.1 – 47.4)
	1 – 10 (84)	0.26 (0.12 – 0.88)	0.39 (0.17 – 1.02)	0.19 (0.11 – 0.45)	0.02 (0.01 – 0.05)	26.0 (16.0 – 43.0)
	11 – 20 (18)	0.27 (0.09 – 0.69)	0.40 (0.13 – 1.29)	0.18 (0.10 – 0.49)	0.02 (0.01 – 0.04)	33.5 (18.9 – 113.7)
	21 – 30 (8)	0.23 (0.13 – 1.11)	0.27 (0.18 – 0.34)	0.15 (0.11 – 0.21)	0.02 (0.01 – 0.04)	30.1 (19.4 – 47.3)
Number of infections during the last 12 months	None (19)	0.60 (0.16 – 4.02)	0.49 (0.10 – 2.24)	0.21 (0.10 – 0.58)	0.02 (0.01 – 0.08)	28.8 (15.9 – 58.7)
	1 to < 5 (247)	0.31 (0.13 – 0.94)	0.49 (0.18 – 1.39)	0.22 (0.11 – 0.48)	0.02 (0.01 – 0.06)	26.2 (10.7 – 46.7)
	5–10 (57)	0.29 (0.13 – 0.79)	0.53 (0.19 – 2.21)	0.23 (0.11 – 0.70)	0.02 (0.01 – 0.04)	27.9 (16.0 – 47.8)
	>10 (7)	0.25 (0.16 – 0.43)	0.56 (0.34 – 0.87)	0.21 (0.15 – 0.27)	0.02 (0.01 – 0.05)	33.4 (26.2 – 48.5)

The concentrations of DDE, ΣPCBs (sum of PCBs), and HCB were all correlated (Table 3). However, we used categorized levels of OC, which were then only marginally correlated; the highest rank correlation was for the PCB and HCB groups (r_Spearman _= 0.46). These correlations did not result in multicollinearity since the tolerance (variance of OC not explained by other predictors) was at least 53%. The volume-based organochlorine concentrations were only marginally correlated with the lipid serum levels. To adjust for lipid concentrations, we included lipid concentrations as a confounder in the explanatory models for leukocyte subsets and immunoglobulins. Results derived from models using the sum of triglycerides and cholesterol compared to triglycerides and cholesterol as individual variables did not reveal any substantial difference (data not shown). We therefore reported results from models using the sum of triglycerides and cholesterol.

**Table 3:** Spearman correlation coefficients between organochlorine compounds (wet-based and lipid-based, n = 331) and their geometric means.

	ΣPCBs	HCB	γ-HCH	Lipids ψ	Lipids §	DDE/lipid	ΣPCBs/lipid	HCB/lipid	γ-HCH/lipid	Geo-metric mean
DDE (μg/L)	0.61 p < 0.01	0.55 p < 0.01	0.16 p < 0.01	0.08 p = 0.09	0.06 p = 0.25	0.86 p < 0.01	0.51 p < 0.01	0.46 p < 0.01	0.14 p < 0.01	0.32
ΣPCBs (μg/L)		0.76 p < 0.01	0.04 p = 0.40	0.04 p = 0.65	0.05 p = 0.34	0.59 p < 0.01	0.90 p < 0.01	0.70 p < 0.01	0.09 p = 0.11	0.50
HCB (μg/L)			0.07 p = 0.19	0.03 p = 0.63	0.04 p = 0.47	0.54 p < 0.01	0.74 p < 0.01	0.83 p < 0.01	0.09 p = 0.11	0.22
γ-HCH (μg/L)				0.15 p = 0.01	0.11 p < 0.05	0.13 p = 0.02	0.05 p = 0.34	0.03 p = 0.64	0.88 p < 0.01	0.02
DDE, lipid-based Ψ (ng/g)							0.63 p < 0.01	0.63 p < 0.01	0.23 P < 0.01	103.03
ΣPCBs, lipid-based Ψ (ng/g)								0.81 p < 0.01	0.14 p = 0.01	164.99
HCB, lipid-based Ψ (ng/g)									0.16 p < 0.01	70.72
HCH, Lipid-based Ψ (ng/g)										6.65

Ψ total lipids calculated as sum of cholesterol and triglycerides

§total lipids calculated using formula 2 of Phillips et al.[28].

rank correlation between total lipids from both formulae was (r_Spearman _= 0.95).

ΣPCBs sum of PCB congeners 101, 118, 138, 153, 170, 180, 183, 187

Regarding lead in whole blood, we found weak correlations with whole blood levels of OC (DDE: *r *= 0.15, *n *= 331, *p *< 0.01; HCB: *r *= 0.14, *n *= 331, *p *< 0.01; γ-HCH: *r *= -0.02, *n *= 331, *p *< 0.70; ΣPCBs: *r *= 0.14, *n *= 331, *p *< 0.01)

Increased white blood cell count (WBC; total leukocytes) was evident in the group with highest DDE level, whereas Pb, at the second, along with PCB at the highest level was associated with a reduction in WBC count. An increase in the number of eosinophils – a leukocyte subset – was identified in the highest DDE category, but not statistically significant, (see Additional file 3). However, eosinophilic granula content was significantly reduced at the upper DDE levels. In addition, IgE count on basophils was increased at higher DDE exposure, being statistically significant for the 0.3–0.43 μg/L category.

Regarding lymphocytes and specific lymphocyte subsets (B-cells, T-cells), the number of T-cells (CD3+), cytotoxic T-cells (CD8+) and B-cells (CD19+) were all significantly reduced in the median Pb category (see Additional file 1). Both natural killer (NK) cells (CD56+) and a NK cells subset (CD57+) were significantly associated with γ-HCH. However, these associations did not reveal dose-dependency.

All four immunoglobulins were associated in a virtually dose dependent fashion to either DDE, HCB or PCBs (see Additional file 2). IgM serum levels increased with the concentration of PCBs (F-test, p < 0.01) but decreased with increasing concentration of HCB (F-test, p < 0.01). In the two upper quartiles of DDE exposures, IgA levels were significantly higher, but lower in the upper quartile of HCB. DDE was not associated in a dose-response mode with IgG (F-test, p = 0.14), however, compared to the reference, the highest DDE exposure group showed a significantly elevated IgG level (t-test, p = 0.04). IgE levels more than doubled as DDE concentration increased (F-test, p = 0.02). The Pb serum levels were related to a significant differences in IgE (F-test: *p *= 0.028), but not in a dose dependent fashion (see Additional file 2).

**Figure 2:**
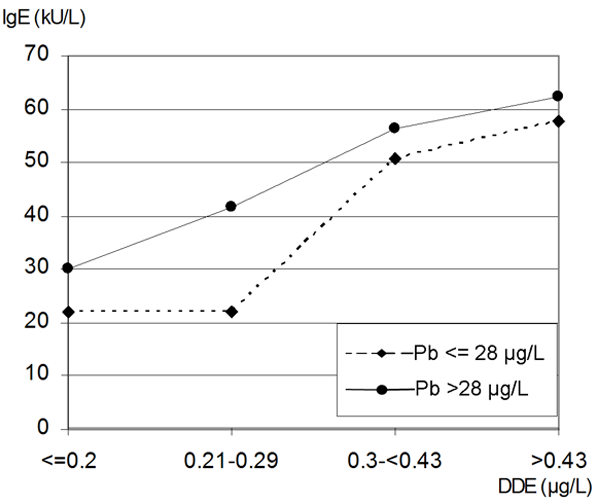
The combined effect of increasing DDE and lead (Pb) on IgE serum levels

Figure 2 shows that both DDE and lead were associated with higher serum IgE levels in children. In groups with lower DDE blood concentrations, Pb concentrations above the median (28 μg/L) were related to increase IgE levels. In groups with higher DDE, there was no additional effect of Pb. Statistically, the combined effect of DDE and Pb on IgE was not significant.

In order to determine whether breastfeeding confounded the associations identified in linear regression models (Figure 1), we repeated our analyses using structural model (path analysis) for exposures determined as significant in linear regressions. Inclusion of breast feeding did not substantially change our findings.

## Discussion

In 331 school children, age 7–10 years, we demonstrated significant relationships between OC and Pb whole blood concentration and cellular and humoral immune markers. First, modest associations were found between NK cells (CD3-CD16+CD56+) and a subset of natural killer cells (CD3-CD16+CD56+CD57+) and γ-HCH (see Additional file 1). Second, HCB was inversely related to IgM. Third, ΣPCBs were directly related to IgM. Fourth, our data showed that Pb decreased the count of T-cells (CD3+), cytotoxic T-cells (CD3+CD8+), and B-cells (CD3+CD5+ CD19+). This reduction was most evident at the 22.1 – 28.3 μg/L Pb concentration, though not in a dose response fashion. Lastly, DDE was inversely related to all immunoglobulins, except IgM (see Additional file 2). However, DDE was not associated with total serum protein (data not shown). The DDE effect was strongest for IgE – more than twofold increase – which also corresponded to an increased count of IgE on basophils. We did not detect a significant relationship between DDE and eosinophils, nevertheless, the number of eosinophils was positively correlated with IgE (*r*_Spearman _= 0.4, *p *< 0.01). However, high DDE levels were found to be significantly associated with lower eosinophilic granula content. The granula contains basic proteins which are cytotoxic and part of the inflammatory response [27].

The cross-sectional nature of the study limits conclusions on whether exposure occurred before immune responses. We can assume that organochlorine concentrations do not vary substantially in childhood, post breastfeeding. There is a decline of PCBs and HCB with age (Table 2), however the assumption of the stability is supported by a follow-up of the same children and OC determined in 1997. The Spearman rank correlation between the two successive measurements were high, with the exception of γ-HCH: DDE: *r *= 0.86, *n *= 274, *p *< 0.01; HCB: *r *= 0.74, *n *= 274, *p *< 0.01; γ-HCH: *r *= 0.1, *n *= 270, *p *= 0.11; ΣPCBs: *r *= 0.82, *n *= 274, *p *< 0.01.

The reported concentrations for OC were not lipid-based. In this cohort, there is a high correlation between lipid- and non lipid-based concentrations for OC (Table 3). Thus, our findings are independent of lipid- or wet weight-based determinations. In our models we controlled for lipids instead of dividing the concentration of OC by the lipid concentration for three reasons. First, a simple division assumes a monotonous linear relation between lipids and organochlorines. Although Phillips and co-workers reported for 20 adults that division by lipids reduces the difference between fasting and non-fasting concentration of OC [28], there is no data to justify a linear relation. Our data in children showed only weak correlations between OC and the sum of cholesterol and triglycerides (Table 3). This correlation did not increase when the sum of lipids were derived by using the 2^nd ^formula proposed by Phillips et al. [28]. Second, there is no standard approach to adjust concentrations below the limit of detection for lipids. In particular, the probability of detection may be influenced by the individual lipid concentration of a child. Third, division by lipids does not take into account that they may confound the organochlorine – immune response relationships. Confounding is likely since lipids and OC are correlated, plus lipids are, for example, associated with the count of lymphocytes [29,30].

There is evidence that breast milk is a significant source of OC, Pb [18-21], and passive immunity [22-24]. Path analytical techniques (Figure 1) were used to verify whether breastfeeding as an intervening variable confounded our associations. The inclusion of breastfeeding in the path analysis did not reveal results different from the linear regression models. Hence, the associations between pollutants and immune markers were independent of breastfeeding.

We found whole blood concentrations of OC in our cohort comparable to similar children in Germany [31]. Compared to children in the United States, age 12–19 years (NHANES – 1999–2000) [32], our DDE values were lower though still within the 95% confidence interval. However, when comparing our results (in whole blood) with those of NHANES (in serum), we have to consider differences between serum and whole blood concentrations. Mes et al. reported that DDE was higher in sera and plasma than in whole blood samples [33]. Conversely, PCBs were higher in whole blood samples. No other comparison with NHANES data was possible as the values for PCB congeners and other OC were below the limit of detection.

Regarding lead (Pb), the geometric mean of 27 μg/L in our investigation was similar to the 33 μg/L found in a study of 797 East-German children 5–14 years of age [34]. Against that, the 1999–2000 NHANES study showed a lower geometric mean (15.1 μg/L) in 905 children 6–11 years of age [32]. However other studies in areas of higher exposure, reported average concentrations above the NHANES value: 40 μg/L for children, 6 to 15 years of age in four communities with mining and smelting operations and two control groups in the United States [6], and 95 μg/L in Chinese children 3–6 years old [8].

We selected a subgroup for blood analyses due to budget constraints. The group having a lower ETS exposure in their homes was selected to reduce the potentially confounding effect of ETS. This group did not significantly differ from other participating children (Table 1). Parents did not know the individual results of the blood analyses, when they provided information on their children, thus reducing recall bias.

The inverse association between DDE and the number of infections 12 months prior to the interview is surprising (Table 2). However, in a logistic regression model the number of infections reported did not show a significant protective effect of DDE. Additionally, when infection was eliminated from the models, there were no major changes in the OC – immune markers association.

The few existing studies estimating the immunotoxicity of lead (Pb) in children, measured by immune markers, are inconsistent in their findings. Regarding immunoglobulins, our positive relation between Pb and IgE was consistent with that of Lutz et al. [7]. However, Sun and co-workers had different results [8]. Concerning lymphocytes, we found that the number of B-cells was significantly reduced with increased Pb concentration. Conversely, Sarasua et al. reported an increase in the number of B-cells for children less than 3 years old [6].

Studies assessing the relation between organochlorine and immune markers, determined in our study, also showed conflicting results and focused mostly on adults [35-37]. In comparison with these adult studies, Vine et al. reported similar modest findings for immunoglobulins and DDE. However, only results for IgA showed statistical significance [35]. Our findings regarding IgE and eosinophilic granula suggest that DDE shifts the immune response into a T helper (Th) 2 direction [38]. Mechanistically, immune responses have been polarized into Th1 and Th2 reactions. Th1 responses lead to the secretion of immunoglobulin G (IgG) and removal of the allergen. The allergic Th2 phenotype is characterized by secretion of cytokines that promote immunoglobulin E (IgE) production resulting in allergies. This suggestion is in agreement with findings of Daniel and co-workers, who reported an association between DDE and interleukin-4, a Th2 cytokine [39]. In addition, our interpretation that DDE may be associated with an allergy-like response is supported by the distribution of aeroallergen-specific IgE results over the four DDE exposure levels. In the lowest DDE exposure group 11.3% of the children showed a positive specific IgE, 10.9% and 12.2% in the two intermediate groups, but 23.0% in the highest DDE exposure group (p = 0.03).

Interestingly, the effects of lead (Pb) and DDE on IgE seems to be competitive. At lower DDE exposure, Pb seems to increase IgE concentrations (Figure 2). There was no additional effect of the other pollutant if one is high; therefore it is possible that both pollutants are involved in the same mechanism. Indeed, studies have surmised that Pb may also shift the immune responses in a Th2 direction [40-42].

There are only few studies on OC blood/serum concentration and immune responses in children. Weisglas-Kuperus et al. reported that prenatal PCB exposure was associated with an increase in the T-cell markers CD3CD8+ and CD4+CD45RO+ [2]. Our data did not support these findings. In another study with prenatal exposures to PCBs, HCB, and DDE, Dewailly et al. did not identify significant associations with immune markers including CD3+, CD4+, CD8+ lymphocytes nor with IgA, IgG, and IgM [3]. However, we found significant relationships between PCBs and HCB with IgM (see Additional file 2). Reichrtova et al. have shown that in utero exposure to DDE is positively correlated with cord serum IgE [43]. No other study of children has investigated the relationship between DDE determined postnatally and Th2 markers such as IgE and eosinophilic granula. This is the second publication showing an association between DDE and serum IgE [4] and the first to report associations between Pb, and DDE and IgE count on basophils and eosinophilic granula.

## Conclusion

In conclusion, our study suggests a non-linear association between IgE and Pb concentration. Regarding OC, our data indicated an increase of IgE related to DDE serum concentrations. A parallel association between DDE, IgE count on basophils, and reduction of eosinophilic granula contents further supports a potential stimulation of a Th_2 _response related to DDE exposure.

Prospective studies should determine more than one OC in a scenario with multiple exposures in order to prevent spurious correlations and include repeated determinations of immune responses to determine changes in immune development during childhood. Furthermore, studies are warranted that determine allergic susceptibilities following DDE and Pb exposure in children.

## List of abbreviations

DDE, dichlorodiphenyl dichloroethene

EDTA, ethylene diamine tetra acetate

ETS, environmental tobacco smoke

FACS, Fluorescence-activated Cell Sorter

FITC, fluorescein isothiocyanate

GC/MS, gas chromatography-mass spectroscopy

HCB, hexachlorobenzene

HCH, hexachlorocyclohexane

HRGC, high resolution gas chromatography

Ig, immunoglobulin

NK-cells, natural killer cells

OC, organochlorine compounds

PCB, polychlorinated biphenyls

PE, Phyoerythrin

PE-Cy5, tandem fluorochrome of PE and cyanine 5

Th1, T-helper 1 cells

Th2, T-helper 2 cells

WBC, white blood cells

## Competing interests

The author(s) declare that they have no competing interests.

## Authors' contributions

WK designed the study and developed the analytical approach. Data analyses and manuscript preparation were done by WK and KRB. TN conducted cell analyses and helped to interpret the findings. HK was responsible for the determinations of organochlorine and lead and revised the manuscript. NOO and JW helped develop the surveys, supported their implementation, and revised the manuscript. All authors approved the final manuscript.

## Supplementary Material

Additional File 3 Table 4: White blood cell, eosinophilic characteristics, and basophilic surface IgE by OC and Pb (geometric mean) Click here for file

Additional File 1 Lymphocyte phenotypes by whole blood DDE, PCBs, HCB, γ-HCH and Pb concentration (geometric mean). The geometric mean of lymphocytes for different levels of OC is presented. Both F- and significant t-tests are also shown. Click here for file

Additional File 2 Immunoglobulins by whole blood DDE, PCBs, HCB, γ-HCH and Pb concentration in children (geometric mean). The geometric mean for different immunoglobulins at different levels of OC is presented. Both F- and significant t-tests are also shown. Click here for file
