# Supplementary material for: Immune function biomarkers in children exposed to lead and organochlorine compounds: a cross-sectional study
Source: Environ Health. 2005 Apr 14;4:5. doi: 10.1186/1476-069X-4-5 (PMC1097747; doi:10.1186/1476-069X-4-5)
Supplement: Additional File 3 — Table 4: White blood cell, eosinophilic characteristics, and basophilic surface IgE by OC and Pb (geometric mean) [file 1476-069X-4-5-S3.pdf]

**Table 4: White blood cell, eosinophilic characteristics, and basophilic surface IgE by OC and Pb (geometric mean)**

| | DDE (μg/L) | | | | Sum of PCBs (μg/L) | | | | HCB (μg/L) | | | | γ-HCH (μg/L) | | | Pb (μg/L) | | | |
|---|---|---|---|---|---|---|---|---|---|---|---|---|---|---|---|---|---|---|---|
| Outcome | ≤ 0.2 | 0.21-0.29 | 0.30-0.43 | >0.43 | ≤ 0.30 | 0.31 - 0.48 | 0.49-0.75 | >0.75 | ≤ 0.15 | 0.16 - 0.2 | 0.21-0.27 | >0.27 | 0.01 | 0.02 | >0.02 | <22.0 | 22.1-28.3 | 28.4-34.1 | >34.1 |
| N | 78 | 89 | 79 | 85 | 80 | 86 | 82 | 83 | 84 | 77 | 86 | 84 | 91 | 130 | 110 | 82 | 81 | 86 | 82 |
| White blood cells, x10$^3$/μL | | | | | | | | | | | | | | | | | | | |
| Total‡ crude | 8136 | 8579 | 8131 | 8555 | 8373 | 8456 | 8528 | 8074 | 8456 | 8354 | 8383 | 8233 | 8062 | 8512 | 8421 | 8612 | 7907 | 8376 | 8552 |
| Adjusted § | 7782 | 8275 | 7970 | 8564* | 8354 | 8318 | 8447 | 7488* | 8260 | 8037 | 8141 | 8131 | 7920 | 8264 | 8247 | 8397 | 7730* | 8167 | 8290 |
| | F-test: p=0.09 | | | | F-test: p=0.02 | | | | F-test: p=0.92 | | | | F-test: p=0.09 | | | F-test: p=0.33 | | | |
| Eosinophil cell count, x10$^3$/μL | | | | | | | | | | | | | | | | | | | |
| Total‡ crude | 162 | 206 | 188 | 207 | 176 | 182 | 197 | 210 | 192 | 187 | 193 | 190 | 186 | 206 | 177 | 176 | 166 | 210 | 214 |
| Adjusted § | 176 | 220 | 208 | 223 | 181 | 194 | 217 | 235 | 245 | 217 | 196 | 172 | 199 | 225 | 194 | 190 | 185 | 233 | 218 |
| | F-test: p=0.37 | | | | F-test: p=0.61 | | | | F-test: p=0.31 | | | | F-test: p=0.32 | | | F-test: p=0.22 | | | |
| Eosinophilic granula | | | | | | | | | | | | | | | | | | | |
| Total‡ crude | 902 | 903 | 901 | 893 | 897 | 901 | 896 | 904 | 899 | 897 | 905 | 897 | 906 | 896 | 898 | 890 | 904 | 900 | 904 |
| Adjusted § | 919 | 913 | 910 | 895* | 902 | 907 | 905 | 923 | 908 | 907 | 916 | 906 | 915 | 906 | 906 | 898 | 913 | 911 | 914 |
| | F-test: p=0.22 | | | | F-test: p=0.37 | | | | F-test: p=0.65 | | | | F-test: p=0.47 | | | F-test: p=0.27 | | | |
| IgE count on basophils | | | | | | | | | | | | | | | | | | | |
| Total‡ crude | 810 | 822 | 863 | 859 | 807 | 842 | 838 | 866 | 808 | 841 | 839 | 867 | 845 | 838 | 833 | 836 | 811 | 845 | 860 |
| Adjusted § | 851 | 851 | 896* | 884 | 860 | 884 | 861 | 877 | 852 | 879 | 866 | 886 | 880 | 868 | 863 | 871 | 845 | 878 | 888 |
| | F-test: p=0.67 | | | | F-test: p=0.50 | | | | F-test: p=0.41 | | | | F-test: p=0.60 | | | F-test: p=0.10 | | | |

‡ absolute number of cells/μL.

* $p \leq 0.05$ based on a t-test compared with the lowest exposure category as the reference.

§ Adjusted for all exposures in the table (OC & Pb) in addition to gender, age, number of infections in the last 12 months, passive smoke exposure in the child's home in the last 12 months, and lipids (sum of cholesterol and triglycerides)
