# Supplementary material for: Immune function biomarkers in children exposed to lead and organochlorine compounds: a cross-sectional study
Source: Environ Health. 2005 Apr 14;4:5. doi: 10.1186/1476-069X-4-5 (PMC1097747; doi:10.1186/1476-069X-4-5)
Supplement: Additional File 1 — Lymphocyte phenotypes by whole blood DDE, PCBs, HCB, γ-HCH and Pb concentration (geometric mean). The geometric mean of lymphocytes for different levels of OC is presented. Both F- and significant t-tests are also shown. [file 1476-069X-4-5-S1.pdf]

Cells	DDE (µg/L)				Sum of PCBs (µg/L)				HCB (µg/L)				γ-HCH (µg/L)			Pb (µg/L)			
	≤ 0.2	0.21-0.29	0.30-0.43	>0.43	≤0.30	0.31 -0.48	0.49-0.75	>0.75	≤0.15	0.16 -0.2	0.21-0.27	>0.27	0.01	0.02	>0.02	<22.0	22.1-28.3	28.4-34.1	>34.1
N	78	89	79	85	80	86	82	83	84	77	86	84	91	130	110	82	81	86	82
T-cells (CD3+)																			
Total‡ crude	2193	2183	2139	2242	2286	2179	2144	2156	2289	2170	2155	2146	2131	2212	2213	2318	2101	2160	2184
Adjusted §	1950	2005	1980	2076	2092	1998	1990	1932	2082	1975	1986	1968	1967	2020	2021	2118	1919*	1979	1999
	F-test: p=0.71				F-test: p=0.74				F-test: p=0.76				F-test: p=0.77			F-test: p=0.17			
T-helper cells CD3+CD4+)																			
Total ‡ crude	1204	1209	1205	1251	1276	1212	1198	1189	1293	1190	1209	1180	1155	1232	1255	1297	1182	1203	1192
Adjusted §	1087	1138	1147	1200	1183	1145	1141	1100	1220	1113	1139	1100	1101	1150	1176	1214	1106	1128	1123
	F-test: p=0.51				F-test: p=0.83				F-test: p=0.37				F-test: p=0.37			F-test: p=0.24			
Cytotoxic T-cells (CD3+CD8+)																			
Total‡ crude	753	743	727	778	773	740	739	752	769	744	740	751	750	753	748	799	711	746	750
Adjusted §	660	665	649	692	697	660	668	642	678	658	662	669	671	669	659	712	634*	661	662
	F-test: p=0.78				F-test: p=0.80				F-test: p=0.97				F-test: p=0.93			F-test: p=0.23			
Memory T-helper cells (CD4+CD45RO+)																			
Total‡ crude	315	249	352	325	316	277	308	325	265	349	311	307	331	274	325	317	304	327	341
Adjusted §	332	323	363	362	343	342	347	347	354	363	345	317	348	341	345	358	321	348	351
	F-test: p=0.18				F-test: p=1.00				F-test: p=0.34				F-test: p=0.91			F-test: p=0.22			
Natural killer cells (CD16+CD56+)																			
Total‡ crude	384	367	350	372	382	378	350	364	369	362	394	348	328	395	373	371	348	365	389
Adjusted §	371	338	334	362	378	366	317	345	326	348	390	342	322	368*	305*	350	322	354	378
	F-test: p=0.48				F-test: p=0.24				F-test: p=0.16				F-test: p<0.01			F-test: p=0.20			
Natural killer cells subset (CD16+CD56+CD57+)																			
Total‡ crude	156	153	148	148	159	156	142	147	155	144	164	142	137	162	151	154	148	148	156
Adjusted §	168	157	158	162	177	171	142	156	148	153	183	162	143	175*	167	162	152	161	169
	F-test: p=0.89				F-test: p=0.21				F-test: p=0.18				F-test: p=0.03			F-test: p=0.69			
B-cells (CD3+CD5+CD19+)																			
Total‡ crude	457	474	456	468	463	461	464	469	473	482	458	446	445	474	469	505	423	462	469
Adjusted §	378	398	382	393	369	372	395	416	420	411	375	349	371	395	397	418	353*	389	393
	F-test: p=1.00				F-test: p=0.67				F-test: p=0.26				F-test: p=0.53			F-test: p=0.10			

‡ absolute number of subtype cells/µL based on percent of lymphocytes x total lymphocyte count.

* $p \leq 0.05$ based on a t-test compared with the lowest exposure category as the reference.

§ Adjusted for all exposures in the table (OC & Pb) in addition to gender, age, number of infections in the last 12 months, passive smoke exposure in the child's home in the last 12 months and lipids (sum of cholesterol and triglycerides)
