# Supplementary material for: Immune function biomarkers in children exposed to lead and organochlorine compounds: a cross-sectional study
Source: Environ Health. 2005 Apr 14;4:5. doi: 10.1186/1476-069X-4-5 (PMC1097747; doi:10.1186/1476-069X-4-5)
Supplement: Additional File 2 — Immunoglobulins by whole blood DDE, PCBs, HCB, γ-HCH and Pb concentration in children (geometric mean). The geometric mean for different immunoglobulins at different levels of OC is presented. Both F- and significant t-tests are also shown. [file 1476-069X-4-5-S2.pdf]

	DDE (µg/L)				Sum of PCBs (µg/L)				HCB (µg/L)				γ-HCH (µg/L)			Pb (µg/L)			
Outcome	≤0.2	0.21-0.29	0.30-0.43	>0.43	≤ 0.30	0.31-0.48	0.49-0.75	>0.75	≤ 0.15	0.16-0.2	0.21-0.27	>0.27	0.01	0.02	>0.02	<22.0	22.1-28.3	28.4-34.1	>34.1
N	78	89	79	85	80	86	82	83	84	77	86	84	91	130	110	82	81	86	82
IgG (mg/dL)																			
Crude	1121	1116	1102	1159	1168	1105	1103	1125	1159	1147	1090	1106	1121	1111	1144	1122	1112	1151	1112
Adjusted §	1177	1197	1199	1295*	1229	1187	1217	1232	1246	1266	1196	1160	1221	1204	1224	1210	1214	1241	1201
	F-test: p=0.14				F-test: p=0.78				F-test: p=0.29				F-test: p=0.84			F-test: p=0.83			
IgA (mg/dL)																			
Crude	128	136	140	134	148	135	124	133	149	138	128	124	139	135	131	129	126	139	146
Adjusted §	112	125	138*	141*	141	127	119	127	145	134	126	111*	130	131	125	123	121	133	136
	F-test: p=0.05				F-test: p=0.34				F-test: p=0.08				F-test: p=0.71			F-test: p=0.27			
IgM (mg/dL)																			
Crude	122	121	125	123	125	122	115	131	126	129	124	114	123	118	129	125	116	129	120
Adjusted §	148	146	153	147	134	138	144	184*	160	164	151	123*	153	142	150	150	143	153	148
	F-test: p=0.92				F-test: p<0.01				F-test: p<0.01				F-test: p=0.42			F-test: p=0.74			
IgE (kU/l)																			
Crude	23	27	33	51	28	34	28	39	26	32	31	43	30	32	35	31	20	46	45
Adjusted §	30	37	53	80*	48	53	41	45	49	51	43	45	41	49	51	46	30	59	59
	F-test: p=0.02				F-test: p=0.84				F-test: p=0.95				F-test: p=0.59			F-test: p=0.03			

* $p \le 0.05$ based on a t-test compared with the lowest exposure category as the reference.

§ Adjusted for all exposures in the table (OC & Pb) in addition to gender, age, number of infections in the last 12 months, passive smoke exposure in the child's home in the last 12 months and lipids (sum of cholesterol and triglycerides)
